# Imaging of Posterior Interosseous Neuropathy following Distal Biceps Repair: A Report of 3 Cases

**DOI:** 10.1155/2015/508924

**Published:** 2015-12-07

**Authors:** Darren Fitzpatrick, Catherine Petchprapa, Leon Rybak

**Affiliations:** ^1^Department of Radiology, Mount Sinai Medical Center, 1 Gustave Levy Place, New York, NY 10029, USA; ^2^Department of Radiology, NYU School of Medicine, 660 1st Avenue, 1st Floor, New York, NY 10016, USA

## Abstract

Three cases of PIN palsy following biceps repair are presented with clinical and imaging correlation. The imaging findings in these cases will be discussed and the orthopedic literature, as regards possible surgical approaches and technical factors believed to predispose to or prevent this complication, will be reviewed. It is important for radiologists to serve as consultants in these uncommon but sometimes devastating complications, helping to quickly and accurately recognize the imaging findings corresponding to the clinical symptoms and aiding the surgeon in diagnosis and treatment by identifying the possible causes and sites of nerve compression.

## 1. Introduction

The elbow is a complex anatomical region, with multiple fine structures in close proximity working in concert to result in efficient function of the upper extremity. Radiologists interpreting MRI of this joint must be well versed with these relationships. Injury to the posterior interosseous nerve (PIN) and its branches is a well-known complication of surgical repair of the distal biceps tendon. Though much has been published in the radiology literature as regards the posterior interosseous nerve syndrome as well as the imaging diagnosis of distal biceps tear, there is lack of literature on imaging of iatrogenic injury to the PIN.

## 2. Cases

### 2.1. Case 1

A healthy, right hand dominant, 51-year-old male sustained a complete rupture of the biceps tendon while shoveling dirt. Within one week of the injury, the patient underwent single incision anterior repair. At the time of surgery, 90% of the biceps tendon was avulsed from the radial tuberosity. The biceps was repaired utilizing a cortical fixation button to secure it to the posterior aspect of the radius.

Immediately following the procedure, the patient complained of increased paresthesia and numbness along the lateral volar aspect of his forearm as well as an inability to extend his wrist (2/5) and fingers (1/5). These findings were attributed to neuropraxia of both the lateral antebrachial cutaneous nerve (LACN) and PIN secondary to operative retraction.

**Figure 1:**
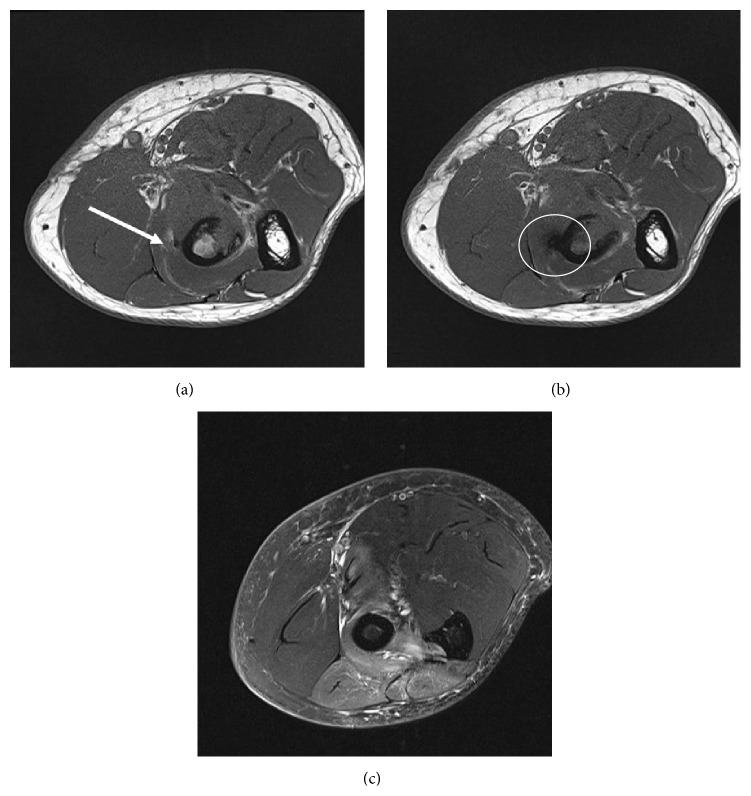
Axial T1 ((a), (b)) and T2 fat saturated images (c) from patient 1. (a) demonstrates the PIN in the supinator tunnel just proximal to the exit point of the radial tunnel posterolaterally where it is clearly identified (white arrow). Slightly more distally, (b), artifact from the cortical fixation button obscures the plane of the PIN (circle). A pattern of muscle edema involving the ECU, EDC, EDM, and supinator muscles is consistent with injury to the PIN in (c).

The patient was placed in an extension splint and was started on active range of motion exercises. When the symptoms failed to resolve in 4-week time, electromyographic (EMG) testing was performed which revealed the expected motor and sensory deficits in the radial nerve and LACN distribution. A magnetic resonance imaging study (MRI) (Figure 1) demonstrated a denervation related edema-like pattern of homogenous high signal on fluid sensitive sequences in the extensor compartment, involving the extensor carpi ulnaris (ECU), extensor digitorum communis (EDC), extensor digiti minimi (EDM), and supinator muscles. No muscle atrophy or fatty infiltration was noted on the T1 weighted images. A surgical scar was noted in the antecubital region with no evidence of a posterior incision. The surgical tunnel within the proximal radius was clearly identified and appeared to be oriented slightly in a proximal to distal direction. In the area of cortical button deployment, along the posteromedial radial cortex, the PIN and corresponding fat plane within the supinator tunnel were focally obscured by small susceptibility artifact. The biceps tendon was not clearly visualized at its insertion prompting the interpreting radiologist to suggest rerupture. However, clinical examination demonstrated intact biceps function and the findings at imaging were attributed to edema and hemorrhage related to the recent postoperative state.

A decision was made to pursue a course of conservative, nonoperative therapy with aggressive physical and occupational therapy. The patient began to recover PIN function by 6 months. At one year, motor function throughout the arm returned to 5/5 with persistent loss of sensation in the LACN distribution.

### 2.2. Case 2

A 44-year-old male presented to the surgeons office with an approximately two-month history of pain along the anterior aspect of the elbow. An MRI was ordered which demonstrated a near-full thickness tear of the distal biceps tendon. Surgery was performed 1 month after presentation and approximately 3 months after the onset of symptoms. At the time of surgery, there was high grade, near-full thickness tear of the tendon just proximal to the insertion site. The torn tendon fibers were retracted to the level of the elbow crease. Repair was performed via a single incision anterior approach with fixation achieved using both cortical fixation button and a biotenodesis screw.

Beginning on postoperative day 4, the patient was noted to have numbness along the dorsal aspect of his thumb, index, and middle digits as well as mild weakness of wrist extension. The patient complained predominantly of loss of function of his thumb. When symptoms failed to resolve with conservative therapy, an EMG was performed which showed evidence of conduction loss in the distribution of both the deep and superficial radial branches.

**Figure 2:**
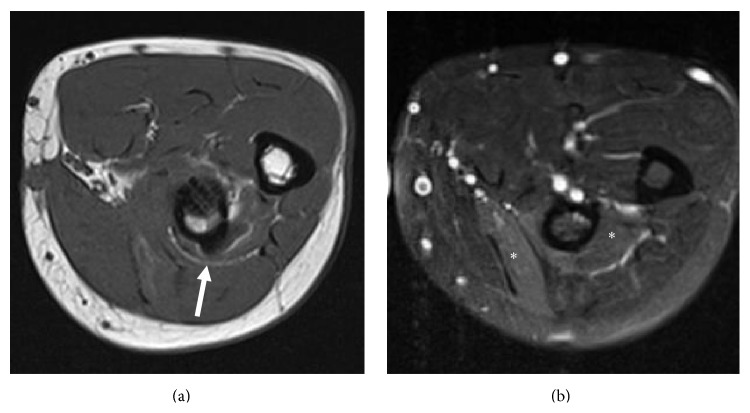
Axial T1 (a) and T2 (b) fat saturated images from patient 2. In (a), surgical artifact along the posterolateral margin of the radius can be seen approaching but not definitively compressing or obscuring the deep radial nerve or proximal PIN (white arrow). Neurogenic edema is noted in the ECRB and supinator muscles in (b) (asterisks).

Approximately 4 months after the surgery and 7 months after the initial injury, a second MRI was performed (Figure 2) which demonstrated an edema-like pattern within the supinator and extensor carpi radialis brevis muscles consistent with denervation. No muscle atrophy was noted. As with the first case, a single anterior scar was identified with no evidence of a posterior incision. Artifact from the area of the cortical fixation button was noted in proximity to the plane of the traversing PIN, but without effacement of the nerve or surrounding fat plane. The biceps repair was intact.

The patient was treated conservatively with physical therapy without resolution of his symptoms. One year later, he opted for nerve exploration and repair at another institution, the details of which are not available. At last contact, he has shown no significant improvement.

### 2.3. Case 3

A 54-year-old, right hand dominant, male sustained a complete rupture of the left distal biceps tendon while playing baseball. The patient underwent surgical repair of the tendon approximately 2 weeks later using a 2-incision approach with suture fixation.

**Figure 3:**
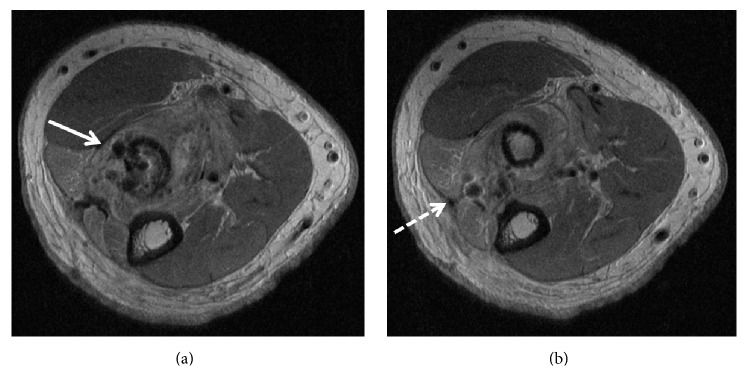
Two sequential axial proton density images ((a), (b)) in patient 3. Susceptibility artifact is noted in the expected location of the PIN along the posterolateral margin of the proximal radius (solid arrow, (a)). Note the scarring posteriorly indicating a two-incision approach (dashed arrow, (b)).

In the immediate postoperative period, the patient reported a burning type pain along the dorsal aspect of his forearm and an inability to extend his fingers. On examination, he had extensor carpi radialis longus function with complete lack of function of the ECU, EPL, and EDC. The consistency of the biceps repair site was noted to be spongy on palpation. A subsequent EMG confirmed the lack of response in the distribution of the PIN. An MRI was performed approximately 1 month after surgery (Figure 3) which demonstrated scattered susceptibility artifact posteriorly along the expected course of the PIN and denervation-like edema signal on fluid sensitive images in all of the muscles of the posterior extensor compartment (supinator, ECU, EDC, and EDM). Mild atrophy and fatty infiltration of the muscles were also noted. Retear of the biceps tendon was identified with proximal tendon retraction of approximately 6 cm. There were surgically created defects in the area of the bicipital tuberosity and adjacent posterolateral radial cortex with no discrete transosseous tunnel. Scarring and susceptibility artifact in the planes between the ECU and EDM muscles posteriorly were consistent with a second posterolateral incision.

Surgical therapies including reexploration and tendon transfer were discussed with the patient who opted instead for conservative treatment consisting of physical therapy and medications. At last visit, the patient has made partial recovery of PIN function but continues to have a significant deficit.

## 3. Discussion

Tears of the distal biceps tendon are relatively uncommon injuries, with most affecting the dominant arm of males in their 40s and 50s. Because conservative treatment may result in a loss of both flexion and supination strength in up to 30% and 40% of cases, respectively, most of these injuries are surgically repaired [1, 2]. The vast majority of patients will return to function; however, complications may occur. Perhaps the most dreaded of these is injury to the posterior interosseous nerve, which can take the form of limited, self-resolving neuropraxia or have devastating implications for function of the hand, requiring tendon transfers or nerve grafting procedures [3]. Though PIN injury can be recognized by classic symptomatology and is treated successfully with conservative therapy, MRI imaging is occasionally necessary to confirm the findings and to elucidate the cause of nerve injury.

Current MRI techniques, with multichannel coils and higher gradients, can accurately image around the surgical artifact produced by distal biceps tendon repair. Metal susceptibility artifact from radial tunnel drilling is usually minimal and does not affect image resolution or obscure the fat planes around the PIN. Bioabsorbable suture anchors in the radial tuberosity also produce minimal artifact. When metallic suture anchors or cortical fixation buttons are placed, the protocol of the MRI exam may be altered to utilize a higher bandwidth, increased echo train length, increased matrix, and increased field of view to maximize visualization of the metallic surgical components and tissue planes adjacent to the PIN.

We have presented three illustrative cases with postoperative imaging of PIN injury sustained at the time of surgical repair of the biceps. In all three cases, a pattern of edema-like signal abnormality was noted within muscles supplied by the PIN, with early atrophy and fatty replacement in one case. This pattern of signal change, which is well defined and confluent and conforms to the boundaries of the specific muscles supplied by a nerve, has a well-documented association with denervation injury. Furthermore, it allows the radiologist to distinguish this injury from other forms of muscle signal abnormality such as the patchy edema seen in postoperative cases such as what is discussed in this report. To further understand the specific imaging findings in these cases, however, it is necessary to be familiar both with the anatomy of the nerve at the elbow and with the different surgical techniques and attendant risks involved in biceps repair.

## 4. Anatomic Considerations

The posterior interosseous nerve is the motor branch of the radial nerve that arises at the level of the elbow and innervates the posterior musculature of the forearm. The nerve gives off small branches to the supinator muscle proximally and has two main terminal branches. The medial or recurrent branch supplies the superficial musculature of the forearm consisting of the extensor digitorum communis (EDC), extensor digiti minimi (EDM), and extensor carpi ulnaris (ECU) while the lateral or descending branch innervates the deep extensors consisting of the abductor pollicis longus (APL), extensor pollicis brevis (EPB), extensor pollicis longus (EPL), and extensor indicis proprius (EIP) [4]. The extensor carpi radialis brevis (ERCB) can be innervated by the PIN as well. The PIN is, thus, responsible for wrist and finger extension and integral to coordinated use of the hand. Depending on the site of injury to the PIN, the patient can present with failure of extension at all metacarpophalangeal (MCP) joints, weakness of thumb abduction and weakness of extension of the wrist with a radial drift (due to the unopposed pull by the unaffected ECRB and extensor carpi radialis longus (ECRL)), loss of extension of the little and ring fingers alone (recurrent branch injury), or loss of extension of the index and thumb and loss of abduction of the thumb alone (descending branch injury) [5].

The PIN originates on average 3-4 cm proximal to the leading edge of the supinator and travels through a space, approximately 3-4 fingerbreadths long, known as the radial tunnel. This tunnel lies along the anterior aspect of the proximal radius with the floor consisting of the radiocapitellar joint capsule proximally and the deep head of the supinator distally. The nerve quickly travels into the posterior forearm, diving deep to the superficial head of the supinator muscle. PIN syndrome, the name given to compression of the nerve with loss of motor function, can be the result of any number of pathologies in this area. Entrapment of the nerve is usually said to occur at 5 common locations: compression by the leading edge of the ECRB, prominent recurrent radial vessels (Leash of Henry), a fibrous leading edge of the supinator tunnel (Arcade of Frohse), within the supinator muscle tunnel itself, or exiting the tunnel. Other etiologies include trauma to the proximal radius, with either acute injury to the nerve or entrapment by subsequent fibrous scarring and compressive masses such as a ganglion cyst, radiobicipital bursa, or distended annular recess of the elbow joint, as can occur with rheumatoid arthritis [4, 5]. Other iatrogenic etiologies include injury during fixation of proximal radial fractures or, as illustrated in these cases, at the time of surgical repair of the biceps tendon.

Knowledge of the course of the radial nerve and PIN and patterns of innervation may suggest the site of compression and branches involved. Based on the distribution of muscle denervation in our cases, involvement of the medial branch of the PIN in case 1 (ECU, EDC, and EDM) and medial (ECU and EDM) and recurrent (EPL) branches in case 3 could be implied. On the other hand, inclusion of the extensor carpi radialis brevis muscle may indicate a more proximal lesion at the level of the radial nerve or variant innervation of the ECRB by the PIN [6].

## 5. Factors Related to Surgical Technique

Surgical techniques of biceps tendon repair have been adapted in part to avoid PIN injury while achieving the strongest and most anatomic repair possible. All techniques require an anterior incision in the antecubital crease to retrieve the biceps tendon. The difference lies in whether an additional posterolateral incision is also utilized. Early reports of biceps repair, involving the classic anterolateral incision first used by Henry, noted a high incidence of injury to the radial, posterior interosseous, and antebrachial nerves. To address this issue, Boyd and Anderson in 1961 suggested use of an anterior incision to retrieve the tendon and a second posterolateral incision to reattach the tendon [7]. This second incision would allow visualization and protection of the radial nerve and PIN during exposure of the radial tuberosity. The 2-incision approach, while decreasing the incidence of PIN injury, has led in some cases to heterotopic ossification (HO) and radioulnar synostosis. More recently, with new available techniques of fixation, including cortical fixation button devices, the single anterior incision approach has experienced resurgence [8].

Further mention of the specific technique of cortical fixation button repair is merited, as this procedure has gained popularity and was performed in the first two cases reported here. With this single anterior incision technique, the tendon edge, attached to a cortical fixation button, can be passed through a tunnel in the radial tuberosity. The cortical fixation button is subsequently deployed on the back side of the tunnel, securing the biceps in place. This repair has been touted as resulting in rapid restoration of strength of the biceps tendon and, thus, early physical therapy and return to activity. It has been criticized, however, for limited exposure to the medially positioned radial tuberosity, thereby preventing accurate reconstruction of the original biceps footprint and resulting in decreased supination strength [9]. It is possible, furthermore, when deploying the cortical fixation button on the posterior tuberosity to trap the PIN between the button and the radius, resulting in nerve damage [10]. Double intramedullary cortical button fixation technique has been shown to be an alternative to the cortical fixation button technique as it has the highest load to failure with theoretical decreased risk of potential PIN injury and more anatomic repair of the biceps footprint because of dual fixation [11].

If information about the surgical technique is not readily available, the radiologist might be able to glean this information by assessing signs of a posterolateral incision. It is likely in these patients that the injury was due to posterior dissection and careful inspection for signs of hemorrhage, scarring, nerve discontinuity, or susceptibility obscuring the PIN, as was seen in case 3, is warranted. Conversely, if a single anterior incision was used, the site of nerve injury is likely at the posterior aspect of the surgically created radial tunnel and may have been sustained at time of tunnel creation or deployment of the fixation devices used (i.e., cortical fixation button). Thus, inspection of the fixation device and its relationship to the PIN should be attempted. In case 2, the PIN was noted in close proximity to the cortical fixation button, whereas in case 1, the nerve was completely obscured by susceptibility artifact in proximity to the device.

The trajectory of the tunnel has been pointed out as critical when performing cortical button fixation. Several groups have demonstrated that creation of the tunnel in 30 degrees of ulnar deviation results in greater distance between the posterior exit site of the tunnel and the PIN [10, 12]. Lo et al. found that tunnel drilling from proximal to distal placed the PIN at the greatest risk [13]. These authors further noted that a small transverse anterior antebrachial incision advocated by some authors may in fact force the surgeon to angle distally and suggested a slightly larger longitudinal incision to allow the surgeon more latitude in tunnel trajectory. The slightly proximal to distal orientation of the tunnel in case 1 may have increased the risk of nerve injury.

## 6. Future Role for Imaging

Most cases of PIN palsy following distal biceps surgery are believed to be temporary in nature and, in many cases, related to stretch of the nerve, pressure due to the pronated position, or inadvertent placement of a retractor in proximity to the nerve. Most of these are treated conservatively and, even in those cases with no resolution, the surgeon may opt to confirm the findings with an EMG and proceed directly to surgical exploration without imaging.

Is there a role for imaging in the prevention, diagnosis, and treatment of this complication? In the preoperative scenario, is it possible that high resolution MRI can help identify normal anatomic variants, which might predispose the patient to this complication. It has been noted that when using a two-incision technique, up to 20% of patients will fail to have complete restoration of supination strength, attributed to disruption of some of the smaller PIN branches to the supinator [13]. Duquin et al. studied the anatomy of the PIN and found 25% of the specimens to have at least 1 branch to the supinator, originating within 5 mm or less of the distal margin of the bicipital tuberosity, and suggested that greater care should be used when dissecting in this area [14]. Other investigators have noted that direct contact of the PIN with the periosteum of the proximal radius, with lack of interposed soft tissue, may place the nerve at greater risk during surgery [15]. With high resolution MR neurography now in use, it might be possible to alert the surgeon to these variants in advance.

Clearly, as indicated in our 3 cases, MRI can identify the pattern of muscle denervation and provide an indirect map of the nerve branches involved. Furthermore, with recent advances in metal artifact reduction MRI techniques, the radiologist can guide therapy by identifying cases where the nerve is transected or entrapped by surgical devices and nothing short of surgical intervention will suffice. Thus, MRI guidance can prevent unnecessary delays, with prolonged periods of conservative therapy, which may doom the patient to permanent deficit [16].

## 7. Conclusion

In conclusion, injury to the radial nerve and its branches, particularly the PIN, is a well recognized complication of surgical repair of the distal biceps tendon that has been described extensively in the orthopedic literature but, to the best of the authors' knowledge, has not been discussed in the radiology literature. Multiple technical factors as well as normal anatomic variations have been identified which increase injury to the PIN during biceps tendon repair. The authors describe 3 cases in which MRI revealed a pattern of marrow edema consistent with neurogenic injury and suggested the site of injury along the course of the PIN. With advancing MRI techniques, including MR neurography and metal suppression, imaging may play an even greater role in the presurgical identification of patients with anatomic variations, which may place them at greater risk for surgical nerve injury as well as identifying the cause of postsurgical nerve damage.
